# Safety, tolerability and pharmacodynamics of apical sodium-dependent bile acid transporter inhibition with volixibat in healthy adults and patients with type 2 diabetes mellitus: a randomised placebo-controlled trial

**DOI:** 10.1186/s12876-017-0736-0

**Published:** 2018-01-05

**Authors:** Renger G. Tiessen, Ciara A. Kennedy, Bradley T. Keller, Nancy Levin, Lisette Acevedo, Bronislava Gedulin, Andre A. van Vliet, Alejandro Dorenbaum, Melissa Palmer

**Affiliations:** 1 Early Development Services, Pharmaceutical Research Associates (PRA) Health Sciences, Van Swietenlaan 6, 9728 NZ Groningen, PO Box 8144, 9702 Groningen, KC Netherlands; 2 Lumena Pharmaceuticals Inc. (part of the Shire group of companies), 12531 High Bluff Drive, Suite 110, San Diego, CA 92130 USA; 3 Lexington, USA

**Keywords:** Apical sodium-dependent bile acid transporter, Bile acids, Clinical pharmacology, Non-alcoholic steatohepatitis, Non-alcoholic fatty liver disease, Phase 1 clinical trial, LUM002, SHP626, Type 2 diabetes mellitus, Volixibat

## Abstract

**Background:**

Pathogenesis in non-alcoholic steatohepatitis (NASH) involves abnormal cholesterol metabolism and hepatic accumulation of toxic free cholesterol. Apical sodium-dependent bile acid transporter (ASBT) inhibition in the terminal ileum may facilitate removal of free cholesterol from the liver by reducing recirculation of bile acids (BAs) to the liver, thereby stimulating new BA synthesis from cholesterol. The aim of this phase 1 study in adult healthy volunteers (HVs) and patients with type 2 diabetes mellitus (T2DM) was to assess the safety, tolerability, pharmacokinetics and pharmacodynamics of ASBT inhibition with volixibat (SHP626; formerly LUM002).

**Methods:**

Participants were randomised 3:1 to receive once-daily oral volixibat (0.5 mg, 1 mg, 5 mg or 10 mg) or placebo for 28 days in two cohorts (HV and T2DM). Assessments included safety, faecal BA and serum 7α-hydroxy-4-cholesten-3-one (C4; BA synthesis biomarker).

**Results:**

Sixty-one individuals were randomised (HVs: placebo, *n* = 12; volixibat, *n* = 38; T2DM: placebo, *n* = 3; volixibat, *n* = 8). No deaths or treatment-related serious adverse events were reported. Mild or moderate gastrointestinal adverse events were those most frequently reported with volixibat. With volixibat, mean total faecal BA excretion on day 28 was ~1.6–3.2 times higher in HVs (643.73–1239.3 μmol/24 h) and ~8 times higher in T2DM (1786.0 μmol/24 h) than with placebo (HVs: 386.93 μmol/24 h; T2DM: 220.00 μmol/24 h). With volixibat, mean C4 concentrations increased by ~1.3–5.3-fold from baseline to day 28 in HVs and by twofold in T2DM.

**Conclusions:**

Volixibat was generally well tolerated. Increased faecal BA excretion and serum C4 levels support the mechanistic rationale for exploring ASBT inhibition in NASH. The study was registered with the Dutch clinical trial authority (Centrale Commissie Mensgebonden Onderzoek; trial registration number NL44732.056.13; registered 24 May 2013).

**Electronic supplementary material:**

The online version of this article (10.1186/s12876-017-0736-0) contains supplementary material, which is available to authorized users.

## Background

In 2014, the World Gastroenterology Organisation estimated that approximately 6 million individuals in the United States (US) had non-alcoholic steatohepatitis (NASH), of whom approximately 600,000 had NASH-related cirrhosis [1]. However, the prevalence of NASH is difficult to establish because definitive diagnosis requires an invasive biopsy for histological confirmation. In a prospective ultrasound study of 400 asymptomatic adults in the US, NASH was confirmed by liver biopsy in 12% of individuals in the overall study population; this value increased to 22% in patients with diabetes mellitus [2]. The authors of this study also reported that 2.7% of patients had evidence of advanced fibrosis (stages 2–4) and, based on this proportion, estimated that more than 2 million middle-aged adults in the US may have undiagnosed advanced NASH.

Non-alcoholic fatty liver disease (NAFLD) ranges from simple steatosis, which is typically non-progressive, to NASH [3], which is characterised by steatosis in addition to inflammation and liver injury (hepatocyte ballooning with or without fibrosis) [4]. Among prospective studies with long-term histological follow-up data (median of at least 3 years between biopsies), progression has been reported in 27–43% of patients with NASH [5–9]. Cirrhosis was identified in more than one-fifth (22%) of individuals with NASH included in a large registry study of liver biopsy specimens (mean follow-up of 8.2 years) [10]. NASH is often associated with type 2 diabetes mellitus (T2DM) [11–13], central or visceral obesity [14], dyslipidaemia [15, 16] and hypertension [17]. Together, these conditions comprise the metabolic syndrome [18–20]. The pathophysiology of NASH is likely to be multifactorial and may include combinations of metabolic, genetic, environmental and microbial gut factors [21].

In the absence of approved pharmacological therapies, treatment of associated metabolic comorbidities, weight reduction, dietary restrictions and incorporation of an exercise routine remain the cornerstones of management [22, 23]. Weight loss, however, is difficult to achieve and maintain [24]. Globally, NASH is a disease with a significant unmet medical need, and its incidence is growing at a rate that parallels that of the obesity epidemic [25]. Indeed, in a recent registry study in the US, the incidence of NASH among adults on the waiting list for a liver transplant increased by 170% between 2004 and 2013 [26]. While this study showed that NASH was the second most common reason for transplantation in 2013 [26], results of a more recent registry analysis indicated that, as of 2014, it had surpassed hepatitis C virus infection as the leading indication for a liver transplant in adults under 50 years of age in the US [27].

Volixibat (SHP626; formerly LUM002) is a highly potent, minimally absorbed, selective inhibitor of the apical sodium-dependent bile acid transporter (ASBT), a transmembrane protein primarily expressed on the luminal surface of ileal enterocytes. ASBT selectively absorbs approximately 60% of excreted bile acids (BAs) in the lumen of the ileum and is critical for intestinal reabsorption of BAs during enterohepatic recirculation (approximately 95% of BAs secreted into bile are recycled back to the liver) [28–30].

Enterohepatic circulation of bile acids between the liver and the intestine plays a central role in facilitating the absorption of dietary lipids, and BAs have important regulatory effects on hepatic lipid and glucose metabolism [31]. Several BA signalling pathways in the liver and intestine have been described, and BAs are known to activate specific nuclear receptors, such as the farnesoid X receptor (FXR), and cell surface receptors, including the G protein-coupled BA receptor TGR5 (also called GPBAR1 or M-BAR) [32, 33]. In the intestine, BAs stimulate the release of peptide hormones, including glucagon-like peptide-1 and -2 (GLP-1 and GLP-2) and peptide YY (PYY), that stimulate insulin secretion [34], inhibit glucagon secretion (glucose dependently) [35], and modulate intestinal growth and function as well as appetite [36]. BAs also stimulate the release of fibroblast growth factors (FGFs), such as FGF-19 and FGF-21 [37], that act in the liver to regulate BA synthesis and glucose and lipid metabolism [31].

Inhibiting ASBT results in a reduction in BA reabsorption, which is associated with an increase in the levels of BAs excreted in the faeces and a decrease in the levels of BAs returning to the liver [38–41]. As assessed by serum levels of 7α-hydroxy-4-cholesten-3-one (C4), an intermediate in the biosynthesis of BAs from cholesterol, an upregulation of BA synthesis in the liver is observed following ASBT inhibition [38–42]. BA synthesis uses low-density lipoprotein cholesterol (LDL-C) as a precursor, and inhibiting enterohepatic reuptake of BAs has been shown to reduce serum LDL-C levels [43]. The role of BAs in both lipid and glucose metabolism has led to the development of ASBT inhibitors as potential pharmacotherapies for hyperlipidaemia [44] and, subsequently, T2DM and the associated metabolic syndrome [45].

Abnormal cholesterol metabolism and accumulation of free cholesterol in the liver have been implicated in the pathogenesis of NASH [46]. Free cholesterol is directly toxic to hepatocytes and is associated with the inflammation and fibrosis observed in this disease [46]. Preclinical studies of ASBT inhibitors have demonstrated positive effects of these agents in NASH-associated conditions: lowering LDL-C and triglyceride levels in mice [42] and guinea pigs [47] fed high-fat diets, and improving glycaemic control in Zucker diabetic fatty rats [45, 48]. In mice fed a high-fat diet, treatment with the ASBT inhibitor SC-435 normalised levels of hepatic triglycerides and serum cholesterol, significantly improved insulin resistance and decreased the NAFLD histological activity score (predominantly steatosis item scores) [49]. In addition, treatment with the ASBT inhibitor resulted in a shift in the serum BA pool from antagonists of FXR to agonists of FXR [49]. Removal of cholesterol from the liver is a potential treatment approach that could decrease, and possibly reverse, damage to hepatocytes. It is postulated that reducing the levels of BAs returning to the liver via inhibition of ASBTs in the terminal ileum may stimulate the liver to synthesise new BA from cholesterol in the liver and serum, and may have therapeutically beneficial metabolic, anti-inflammatory, anti-steatotic and anti-fibrotic effects in patients with NASH [49].

This paper reports the results of a phase 1, placebo-controlled, dose-escalation study of the ASBT inhibitor volixibat in healthy adults and patients with T2DM. The objectives of the study were to evaluate the safety and tolerability, and the pharmacodynamic (PD) and pharmacokinetic (PK) effects, of multiple doses of volixibat administered orally over a 28-day period.

## Methods

### Study design

This was a 28-day, randomised, double-blind (investigators and participants), parallel-group study in healthy volunteers (HVs) and patients with T2DM. For HVs, screening took place from day −21 to day −1 and for patients with T2DM from day −42 to day −16. Patients with T2DM discontinued their anti-diabetic medications starting on day −14. Thereafter, these individuals measured glucose levels once daily, after an overnight fast, up to day 28 to check that their fasting blood glucose levels were below 12.5 mmol/L (225 mg/dL). Patients with T2DM were withdrawn from the study if their fasting blood glucose level reached 12.5 mmol/L on two consecutive days during the treatment period.

**Fig. 1:**
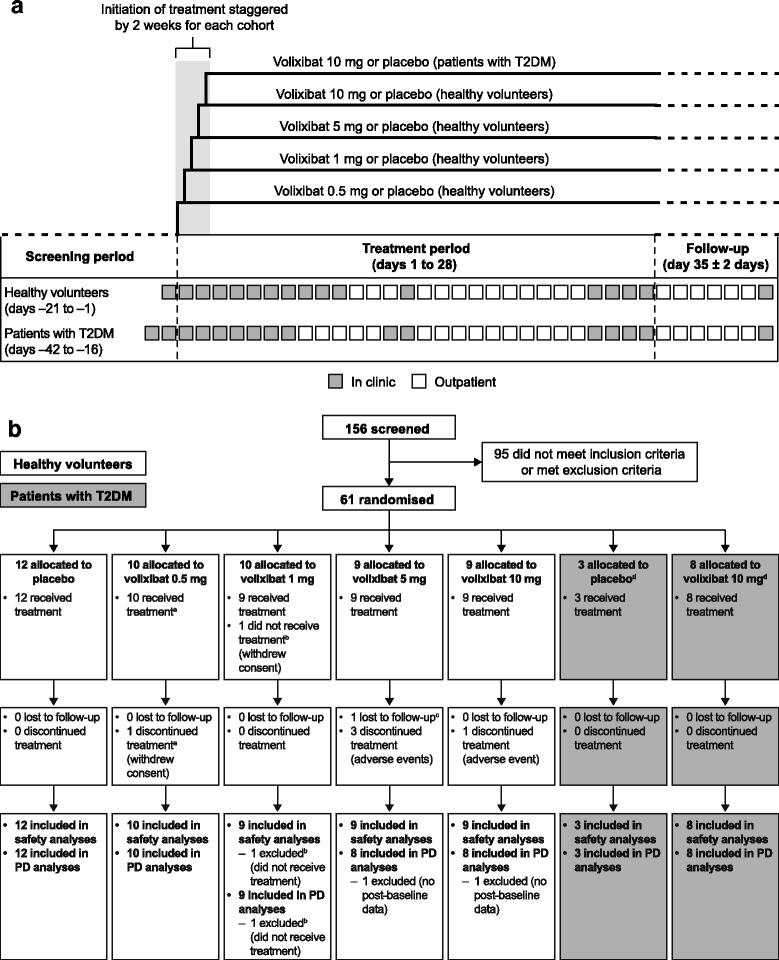
**a** Study design and (**b**) disposition of participants. ^a^One participant withdrew consent on day 19 (last treatment on day 16); an additional participant was randomised as a replacement. ^b^One participant withdrew consent owing to personal reasons between randomisation and treatment initiation on day 1; an additional participant was randomised as a replacement. ^c^Received all planned treatments before being lost to follow-up. ^d^Only 11 of the 12 planned patients with T2DM were enrolled owing to difficulties in recruiting individuals who met all inclusion/exclusion criteria. *PD* pharmacodynamic; *T2DM* type 2 diabetes mellitus

Participants were randomised 3:1 to receive once-daily oral doses of volixibat (0.5 mg, 1.0 mg, 5.0 mg or 10.0 mg) or placebo for 28 days. The group of participants assigned to receive the lowest dose of volixibat (or placebo) in the HV cohort commenced treatment first. Further groups of HVs were started on escalating doses (or placebo) at 2-week intervals (Fig. 1), contingent upon acceptable assessments of safety and tolerability from the preceding dose. In the T2DM cohort, patients began treatment with volixibat 10 mg (or placebo) 2 weeks after the HV cohort. Clinic visit schedules are shown in Fig. 1. A follow-up telephone call was made on day 56 (±3 days).

The study was conducted in compliance with the International Conference on Harmonisation E6 Guideline for Good Clinical Practice (Committee for Proprietary Medicinal Products guideline CPMP/ICH/135/95) and was compliant with the European Union Clinical Trial Directive 2001/20/EC. The clinical study protocol, protocol amendments and informed consent forms were reviewed and approved by an independent ethics committee (Stichting Beoordeling Ethiek Biomedisch Onderzoek, Assen, Netherlands). Participants signed the informed consent forms during the pre-study screening visit, within a period of 3 weeks (HVs) or 6 weeks (patients with T2DM) before the first dosing day and before the start of any study-related procedures. Individuals were screened for eligibility by a contract research organisation (Pharmaceutical Research Associates [PRA] Group BV, Zuidlaren, Netherlands); healthy individuals and patients with T2DM were screened within a period of 3 or 6 weeks, respectively, before first administration of the study drug. The randomisation list was prepared by a statistician at PRA who was not involved in the study; within each dose group, a block size of 12 was used to attain a 3:1 randomisation to volixibat or placebo, respectively. Randomisation codes were provided to a pharmacist at PRA who prepared the medication (capsules containing volixibat or placebo were indistinguishable). The study took place between 21 May 2013 and 8 February 2014 at a single study centre in Groningen, Netherlands, and was registered with the Dutch clinical trial authority (Centrale Commissie Mensgebonden Onderzoek; registration number NL44732.056.13).

### Participants

Individuals eligible to participate in the study were men and women aged 18–70 years (for HVs: 18–55 years inclusive for doses of 0.5 mg, 1 mg and 5 mg, and 45–65 years inclusive for the 10 mg dose; for patients with T2DM: 18–70 years inclusive) with a body mass index (BMI) of 18.0–30.0 kg/m^2^ for HVs and 22.0–35.0 kg/m^2^ for patients with T2DM.

Patients with T2DM had to have a glycosylated haemoglobin (HbA_1c_) level greater than 6.0% and less than 10% at screening, and a fasting blood glucose level of 7.0–12.5 mmol/L (126–225 mg/dL) at entry into the clinical research centre (day −2). Patients with T2DM were also required to have been taking a stable dose of one or more oral anti-diabetic medications (e.g. metformin, sulphonylurea or any other orally administered glucose-lowering drug) for at least 3 months before screening, to have been receiving no other medications, including dietary supplements, which significantly alter blood glucose control, and to be able and willing to wash out all anti-diabetic medication for 14 days before dosing.

HVs were excluded from the study if they had a history of chronic disease. Patients with T2DM were excluded if they had used insulin or thiazolidinediones 3 months before screening, or if they had advanced diabetic complications, including neuropathy, nephropathy and retinopathy. Complete inclusion and exclusion criteria are provided in Additional file 1.

### Outcome measures

The primary objective of this study was to evaluate the safety and tolerability of orally administered volixibat in adult HVs and patients with T2DM. Safety assessments were based on recording of treatment-emergent adverse events (TEAEs), vital signs, 12-lead electrocardiogram analyses, results of clinical laboratory tests, physical examinations and, in the T2DM cohort, blood glucose measurements.

Secondary objectives were to evaluate the PK and PD properties of volixibat, to explore the effect of volixibat on liver enzymes (alanine aminotransferase, aspartate transaminase, γ-glutamyl transferase, alkaline phosphatase and lactate dehydrogenase) and fat absorption parameters (levels of 25-hydroxy vitamin D, β-carotene, retinol, retinol binding protein, tocopherol and total lipids) and to determine the effect of volixibat on glucose metabolism in patients with T2DM.

#### PK assessments

The study aimed to calculate the following PK parameters of volixibat: maximum observed plasma concentration; time of maximum observed plasma concentration; and area under the plasma concentration–time curve (AUC) from time 0 to time t, where time t is the time of the last quantifiable plasma concentration.

#### PD assessments

In the HV cohort, the following PD variables were measured: serum and faecal total BA concentrations (amount excreted per 24 h collection interval), biomarkers of BA synthesis/metabolism [33] (C4, FGF-19 and FGF-21) and lipids (total cholesterol, LDL-C, high-density lipoprotein cholesterol [HDL-C] and triglycerides). In the T2DM cohort, the following additional PD variables were measured: glucose metabolism biomarkers (levels of fasting glucose, insulin, HbA_1c_ and fructosamine) and meal tolerance test (MTT) response (concentrations of glucose, insulin, C-peptide, total GLP-1, GLP-2 and PYY).

### Data analysis

A placebo arm was included in the study design to investigate whether any observed abnormalities were likely to be due to active treatment with volixibat or to the study procedures, and not for a formal comparison between the volixibat and placebo groups. The sample size was based on previous experience and was selected to ensure that safety and tolerability could be assessed adequately while minimising unnecessary exposure to volixibat. On this basis, the study planned to have 12 individuals (volixibat, *n* = 9; placebo, *n* = 3) in each of four groups of HVs and in one group of patients with T2DM, giving a total planned sample size of 60 participants.

All participants who had received at least one dose of study drug and had at least one post-baseline PD assessment were included in PD analyses. PK analyses were carried out on data from all participants who had received volixibat and for whom sufficient bioanalytical data were available with which to calculate reliable estimates of PK parameters. Safety analyses were carried out on data from all participants who had received at least one dose of the study drug.

Statistical analyses were performed using SAS® Version 9.1.3 (SAS Institute Inc., Cary, NC, USA). Safety, PK and PD data were summarised by volixibat dose within each cohort, and safety and PD data were summarised for patients receiving placebo within each cohort using descriptive statistics (number of observations, mean, standard deviation [SD], median, and minimum and maximum values). Categorical data were summarised using frequencies and percentages. For assessment of changes in parameters from baseline, the baseline was taken as the last measurement before the first dose of the study drug; for assessment of changes in glucose parameters from baseline, the baseline was taken as the pre-MTT measurement. Levels of glucose metabolism biomarkers (glucose, insulin, C-peptide, total GLP-1, GLP-2 and PYY) in the T2DM cohort were compared on days 14 and 28 in the volixibat 10 mg and placebo groups in an exploratory analysis of the following PD parameters: pre-MTT concentration (C_pre_); maximum observed change from the pre-MTT baseline measurement (E_max_); AUC from time 0 to 3 h after the MTT (AUC_(0–3)_) calculated using the linear trapezoidal rule; AUC from time 0 to 3 h after the MTT with the baseline value subtracted (rAUC_(0–3)_) and calculated using the linear trapezoidal rule as rAUC_(0–3)_ = AUC_(0–3)_ – (C_pre_ × 3); updated homeostasis model assessment of insulin resistance (HOMA2-IR) [50]; and homeostasis model assessment of β-cell function (HOMA2-%B). These comparisons were performed using a mixed model with treatment, study day and treatment-by-study-day interaction as fixed factors, participant within treatment as a random factor and the day −1 (baseline) value for each participant as a covariate. From this model, the difference in each PD parameter, 90% confidence intervals (CIs) and *P* values were calculated on days 14 and 28. As these exploratory comparisons were not corrected for multiplicity, all *P* values should be considered to be nominal.

## Results

### Participant disposition and demographics

During dose escalation, the safety and tolerability of volixibat were considered to be acceptable at each planned dose level by the principal investigator and the independent ethics committee. Therefore, participants were assigned to receive volixibat up to the maximum planned daily dose of 10 mg. Of the 61 individuals who were randomised, 60 received at least one dose of study drug (49 HVs and 11 patients with T2DM) and were included in the safety population. The PD population included 58 of these participants because two individuals did not have a post-baseline PD assessment. Details are provided in Fig. 1b.

Seven participants discontinued from the study (Fig. 1b): four were withdrawn from the study owing to TEAEs (of which one was a serious TEAE, but this was not considered to be treatment-related), two withdrew consent, and one was lost to follow-up. One HV in the volixibat 0.5 mg group and one in the volixibat 1 mg group who withdrew were replaced with additional participants. Owing to the difficulty in recruiting patients with T2DM who met all the inclusion criteria, only 11 of the 12 participants planned for enrolment in this cohort were randomised (volixibat, *n* = 8; placebo, *n* = 3). In total, 54 participants completed the study according to the protocol.

Baseline participant demographics are summarised in Table 1. Within the HV cohort, variations in age among the five groups were consistent with variations in the age enrolment criteria for each group. The ratio of men to women was approximately 1:1 in the HV cohort, except for in the volixibat 0.5 mg and 5 mg dose groups, in which 20.0% and 22.2%, respectively, were women. There was minimal variation in BMI among members of the HV dose groups, and the majority of participants were Caucasian. All patients in the T2DM cohort were men, and all but one participant were Caucasian. In the T2DM cohort, there were no notable differences in baseline characteristics between the volixibat and placebo groups.

**Table 1:** Demographic and baseline characteristics

Characteristic	Healthy volunteers					Patients with T2DM	
	Placebo	Volixibat				Placebo	Volixibat
	(*n* = 12)	0.5 mg (*n* = 10)	1 mg (*n* = 9)	5 mg (*n* = 9)	10 mg (*n* = 9)	(*n* = 3)	10 mg (*n* = 8)
Age, years	38.9 ± 15.58 (20–62)	28.2 ± 11.65 (19–54)	28.9 ± 10.86 (19–45)	30.2 ± 12.46 (19–54)	59.6 ± 4.72 (55–65)	67.7 ± 2.08 (66–70)	65.5 ± 3.38 (61–70)
BMI, kg/m^2^	23.82 ± 1.816 (21.6–27.4)	23.74 ± 3.301 (20.1–28.7)	24.10 ± 2.841 (20.2–28.9)	23.12 ± 3.423 (19.0–29.1)	22.79 ± 2.958 (18.7–28.8)	29.77 ± 1.914 (28.0–31.8)	29.49 ± 3.512 (24.4–34.8)
Female, *n* (%)	7 (58.3)	2 (20.0)	4 (44.4)	2 (22.2)	5 (55.6)	0	0
Race, *n* (%)							
Caucasian	11 (91.7)	9 (90.0)	7 (77.8)	7 (77.8)	9 (100.0)	3 (100.0)	7 (87.5)
Other	1 (8.3)	1 (10.0)	2 (22.2)	2 (22.2)	0	0	1 (12.5)

*BMI* body mass index, *T2DM* type 2 diabetes mellitus

Values are mean ± standard deviation (range) unless otherwise stated; data are from the safety analysis set

### PK effects

PK parameters could not be calculated because serum concentrations of volixibat were below the lower limit of quantitation (0.05 ng/mL) at all time points in all but one study sample; a patient in the volixibat 10 mg group of the T2DM cohort had a plasma concentration of 0.06 ng/mL at 147 h post-dose.

### Safety and tolerability

Of the 60 participants included in the safety population, study medication was received by 49 HVs (volixibat, *n* = 37; placebo, *n* = 12) and 11 patients with T2DM (volixibat, *n* = 8; placebo, *n* = 3). Dosing for 28 consecutive days was achieved in 55 participants.

#### TEAEs

In total, 240 TEAEs were reported in 43 HVs (87.8%), and 24 TEAEs were reported in 8 patients with T2DM (72.7%) (Table 2). Most TEAEs were mild (HVs, *n* = 214; patients with T2DM, *n* = 17) or moderate (HVs, *n* = 26; patients with T2DM, *n* = 7) in severity, and no deaths occurred. There was no clear relationship between the treatment groups in the frequency of moderate TEAEs.

**Table 2:** Summary of treatment-emergent adverse events

Participants, *n* (%)	Healthy volunteers					Patients with T2DM	
		Volixibat				Placebo	Volixibat
	Placebo (*n* = 12)	0.5 mg (*n* = 10)	1 mg (*n* = 9)	5 mg (*n* = 9)	10 mg (*n* = 9)	(*n* = 3)	10 mg (*n* = 8)
Any adverse event	10 (83.3)	7 (70.0)	9 (100)	8 (88.9)	9 (100)	1 (33.3)	7 (87.5)
Treatment-related adverse events	6 (50.0)	7 (70.0)	8 (88.9)	8 (88.9)	9 (100)	1 (33.3)	6 (75.0)
Severe adverse events	0	0	0	0	0	0	0
Serious adverse events	0	0	0	0	1 (11.1)^a^	0	0
Deaths	0	0	0	0	0	0	0
Adverse events reported in >1 participant in any group^b^							
Gastrointestinal disorders							
Diarrhoea	3 (25.0)	5 (50.0)	8 (88.9)	8 (88.9)	9 (100)	1 (33.3)	6 (75.0)
Abdominal pain	3 (25.0)	3 (30.0)	5 (55.6)	6 (66.7)	7 (77.8)	1 (33.3)	3 (37.5)
Gastrointestinal sounds abnormal	2 (16.7)	0	0	2 (22.2)	4 (44.4)	0	1 (12.5)
Nausea	2 (16.7)	1 (10.0)	1 (11.1)	1 (11.1)	4 (44.4)	0	0
Flatulence	0	1 (10.0)	2 (22.2)	0	4 (44.4)	1 (33.3)	0
Abdominal distension	1 (8.3)	2 (20.0)	1 (11.1)	0	2 (22.2)	0	0
Abdominal discomfort	1 (8.3)	1 (10.0)	2 (22.2)	0	0	0	0
Dry mouth	0	0	0	0	0	0	2 (25.0)
Nervous system disorders							
Headache	4 (33.3)	2 (20.0)	3 (33.3)	1 (11.1)	3 (33.3)	0	1 (12.5)
Dizziness	1 (8.3)	0	0	0	2 (22.2)	0	1 (12.5)
General disorders and administration site conditions							
Fatigue	1 (8.3)	0	2 (22.2)	1 (11.1)	0	0	0
Influenza-like illness	0	0	0	2 (22.2)	0	0	0
Infections and infestations							
Rhinitis	2 (16.7)	1 (10.0)	0	1 (11.1)	0	0	1 (12.5)
Respiratory, thoracic and mediastinal disorders							
Oropharyngeal pain	3 (25.0)	0	1 (11.1)	0	0	0	0
Musculoskeletal and connective tissue disorders							
Pain in extremity	0	0	2 (22.2)	0	0	0	0

*T2DM* type 2 diabetes mellitus

Data are from the safety analysis set

^a^Ablation of the retina with a bleed in the vitreous body of the right eye (moderate severity), which was not considered to be related to the study drug by the principal investigator. ^b^Reported by System Organ Class in descending order of frequency in the volixibat 10 mg group for healthy volunteers

One participant in the HV cohort (10 mg dose group) was withdrawn from the study owing to a serious TEAE (ablation of the retina with a bleed in the vitreous body of the right eye), which was considered by the principal investigator to be unrelated to the study drug. Three participants were withdrawn for non-serious TEAEs. In one of these participants, the TEAE was not considered to be treatment-related (moderate Epstein–Barr virus [EBV] infection; HV cohort, 5 mg dose group). The treatment-related TEAEs were mild diarrhoea accompanied by blood in one individual, and mild diarrhoea and mild anal erosion in another individual (both in the HV cohort, 5 mg dose group). The TEAE of mild diarrhoea accompanied by blood was first reported on day 3 after the third dose of volixibat and resolved without medication 3 days after treatment discontinuation on day 5. In the participant with mild diarrhoea and mild anal erosion, diarrhoea was first reported on day 1 and continued until the patient was withdrawn on day 21; anal erosion was first reported on day 13 and persisted for approximately 1.5 h (the participant reported that they had seen blood on the stools, which came from the anal erosion due to the diarrhoea).

Most TEAEs were transient and resolved before the end of the study; one TEAE of mild oropharyngeal pain was ongoing at follow-up, and three TEAEs were improving. TEAEs considered to be related to the study drug were reported in 38 HVs (77.6%) and 7 patients with T2DM (63.6%); the majority of drug-related TEAEs were categorised as gastrointestinal disorders, which included diarrhoea and abdominal pain (Table 2).

#### Laboratory tests and clinical examinations

With the exception of the participant who developed EBV infection, there were no clinically relevant variations in vital signs. Physical examinations did not identify any abnormalities of clinical relevance. No trends or clinically significant changes in heart rate or rhythm or body weight were observed. In the T2DM cohort, there were no abnormalities in fasting blood glucose levels. Although several individual changes from baseline were observed in the clinical laboratory values, no clinically relevant changes were seen, including those relating to levels of serum electrolytes and serum liver enzymes, and fat absorption parameters.

### PD effects

#### BA uptake and synthesis

**Fig. 2:**
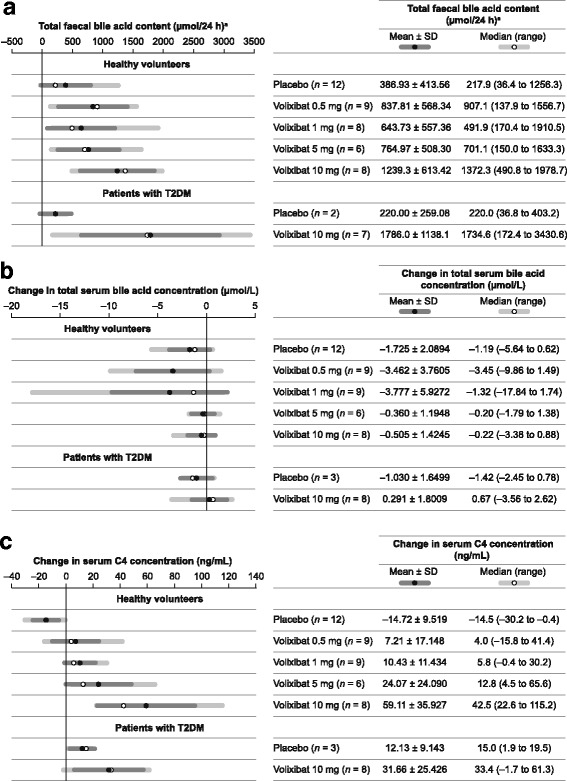
Bile acid uptake and synthesis: (**a**) total faecal bile acid content on day 28 and change from baseline to day 28 in (**b**) total serum bile acid and (**c**) C4 concentration. *n* is the number of participants with available data on day 28. Data are from the pharmacodynamic analysis set. ^a^Determined over 48 h between days 26 and 28. *C4* 7α-hydroxy-4-cholesten-3-one; *SD* standard deviation; *T2DM* type 2 diabetes

Mean total faecal BA excretion was approximately 1.6–3.2 times higher in HVs who received once-daily volixibat for 28 days than in those receiving placebo (Fig. 2a). Mean total BA excretion (± SD) was greatest with volixibat 10 mg (1239.3 ± 613.42 μmol/24 h compared with 386.93 ± 413.56 μmol/24 h in the placebo group); no clear relationship between BA excretion and dose was discernible at doses of 0.5–5 mg. In the T2DM cohort, the mean total BA excretion on day 28 was approximately eight times higher with volixibat 10 mg (1786.0 ± 1138.1 μmol/24 h) than with placebo (220.0 ± 259.08 mol/24 h).

At baseline (day 1), there were no notable differences in fasting serum concentrations of BAs (total), FGF-19 or FGF-21 between the volixibat and placebo groups in either the HV or T2DM cohorts (Table 3). No notable changes from baseline to day 28 in these serum measures were observed, and differences between the volixibat and placebo groups were not considered to be clinically relevant. In the HV cohort, a slight reduction in mean (± SD) fasting total serum BA concentration was observed from baseline to day 28 in all groups, which was most pronounced in the volixibat 0.5 mg (−3.462 ± 3.7605 μmol/L) and 1 mg (−3.777 ± 5.9272 μmol/L) groups (Fig. 2b). No notable dose-related changes were seen in the major serum BA species (cholic acid, chenodeoxycholic acid, deoxycholic acid, ursodeoxycholic acid and lithocholic acid) in the HV or T2DM cohorts.

**Table 3:** Summary of serum bile acids and serum markers of bile acid synthesis/metabolism

	Day	Number	Serum bile acid (μmol/L)		C4 (ng/mL)		FGF-19 (pg/mL)		FGF-21 (pg/mL)	
			Mean	SD	Mean	SD	Mean	SD	Mean	SD
Healthy volunteers										
Placebo	1	12	4.676	3.3066	29.95	12.917	359.2	500.55	174.1	90.84
	14	12	2.941	2.2596	15.21	12.211	264.3	280.50	224.2	180.73
	28	12	2.951	2.3758	15.23	8.537	259.3	333.54	167.6	112.15
Volixibat 0.5 mg	1	10	5.886	4.2363	22.87	6.427	304.8	328.52	195.6	155.49
	14	10	2.304	1.6197	35.57	25.119	297.0	302.87	219.7	133.12
	28	9	2.540	1.9897	30.21	13.358	306.7	247.72	204.6	104.43
Volixibat 1 mg	1	9	5.614	5.7738	14.49	5.304	495.3	969.34	138.9	118.64
	14	9^a^	4.176	3.9589	23.60	13.736	551.1	1129.55	206.6	195.40
	28	9^a^	1.838	0.9931	24.92	14.686	1056.7	2428.36	150.2	99.92
Volixibat 5 mg	1	8	2.826	1.4719	30.78	19.365	669.0	1301.49	224.0	111.03
	14	8	1.861	1.4777	56.38	43.306	651.4	1324.94	252.3	146.26
	28	6	2.872	1.6163	56.52	38.863	784.7	1405.55	215.2	142.22
Volixibat 10 mg	1	8	2.248	1.7120	13.75	4.602	228.1	239.87	204.1	156.90
	14	8	1.959	1.1725	37.51	18.886	186.6	242.72	253.9	165.21
	28	8	1.743	0.9222	72.86	35.499	164.6	226.91	212.6	157.95
Patients with T2DM										
Placebo	1	3^b^	2.370	1.6532	16.60	6.899	211.5	212.84	360.0	300.66
	14	3	2.523	2.2756	14.40	3.568	94.3	121.25	463.0	386.47
	28	3^b^	1.340	0.1758	28.73	8.545	173.5	197.28	309.3	184.02
Volixibat 10 mg	1	8	1.826	1.6175	30.15	14.351	605.8	1264.78	334.0	128.76
	14	8^c^	2.355	1.1042	59.66	28.221	640.1	1399.18	497.9	198.04
	28	8	2.118	0.9359	61.81	22.163	114.4	146.91	359.0	178.13

*C4* 7α-hydroxy-4-cholesten-3-one, *FGF* fibroblast growth factor, *N* number of patients for whom data were available, *SD* standard deviation, *T2DM* type 2 diabetes mellitus

On day 1 (baseline), measurements were taken before participants received the first dose of study drug. Data are from the pharmacodynamic analysis set

^a^Fibroblast growth factor data available for 8 patients on day 14 and 7 patients on day 28. ^b^Fibroblast growth factor data available for 2 patients on day 1 and 2 patients on day 28. ^c^Fibroblast growth factor data available for 7 patients on day 14

Serum C4 concentrations at baseline and on days 14 and 28 are shown in Table 3. In the HV cohort, there was a dose-dependent increase in mean serum C4 concentration from baseline to day 28 in the volixibat groups compared with a decrease in the placebo group (Fig. 2c). At volixibat doses of 0.5–5 mg, mean serum C4 levels on day 14 were approximately 1.5 − 1.8 times higher than baseline and remained similar on day 28 at approximately 1.3–1.8 times higher than baseline; in the 10 mg group, levels were 2.7 times higher than baseline on day 14 and increased further to approximately 5.3 times higher than baseline on day 28 (Table 3). In the placebo group, mean serum C4 levels were half that of baseline on days 14 and 28. In the T2DM cohort, mean (± SD) serum C4 levels in the volixibat group were approximately twofold greater on day 14 (59.66 ± 28.221 ng/mL) and day 28 (61.81 ± 22.163 ng/mL) than at baseline (30.15 ± 14.351 ng/mL) (Table 3). In the placebo group, mean serum C4 levels at baseline (16.60 ± 6.899 ng/mL) were lower than those in the volixibat group, remained similar on day 14 (14.40 ± 3.568 ng/mL) and had increased slightly on day 28 (28.73 ± 8.545 ng/mL) to a level similar to that observed before treatment in the volixibat group.

#### Lipid profile

At baseline, all participants were normolipaemic, and there were no major differences among treatment groups in serum lipid concentrations (total cholesterol, LDL-C, HDL-C and triglycerides) (Table 4). No clinically relevant changes in lipid concentrations from baseline to day 14 or day 28 were observed in either cohort, and there were no clinically relevant differences between the volixibat and placebo groups on days 14 or 28. In the HV cohort, the mean total cholesterol level was slightly lower on day 14 and day 28 than at baseline in the volixibat 0.5 mg, 1 mg and 10 mg groups (Table 4); in the volixibat 5 mg group, there was little variation throughout the study. Similarly, compared with baseline, the concentration of LDL-C was slightly lower on days 14 and day 28 in the volixibat 1 mg and 10 mg groups; in the 0.5 mg and 5 mg groups, no trend was evident between days 1 and 28. Changes from baseline to days 14 and 28 in total cholesterol, LDL-C and triglyceride levels were not clinically significant, but the changes tended to be smaller in the volixibat groups than in the placebo group; no obvious trend in HDL-C changes from baseline was evident between treatment groups (Table 4).

**Table 4:** Summary of blood lipid parameters at baseline and on days 14 and 28

	Day	Number	Total cholesterol (mmol/L)		LDL-C (mmol/L)		HDL-C (mmol/L)		Triglycerides (mmol/L)	
			Mean	SD	Mean	SD	Mean	SD	Mean	SD
Healthy volunteers										
Placebo	1	12	4.70	0.964	2.78	0.853	1.38	0.298	1.189	0.3600
	14	12	4.78	1.209	3.03	1.112	1.29	0.223	1.031	0.3635
	28	12	4.73	1.097	2.87	0.934	1.38	0.322	1.233	0.4302
Volixibat 0.5 mg	1	10	4.17	0.933	2.39	0.861	1.35	0.314	0.976	0.3523
	14	10	4.07	0.686	2.23	0.726	1.31	0.307	1.013	0.3370
	28	9	3.99	0.854	2.29	0.851	1.37	0.274	0.936	0.2013
Volixibat 1 mg	1	9	4.28	0.628	2.43	0.650	1.34	0.274	0.863	0.3278
	14	9	3.87	0.650	2.22	0.655	1.29	0.215	0.962	0.6851
	28	9	3.97	0.552	2.22	0.576	1.33	0.300	1.024	0.5973
Volixibat 5 mg	1	8	4.53	0.865	2.81	0.781	1.25	0.227	1.476	0.6576
	14	8	4.21	0.825	2.50	0.725	1.23	0.276	1.294	0.7138
	28	6	4.58	1.102	2.77	0.967	1.33	0.350	1.522	0.5151
Volixibat 10 mg	1	8	5.44	0.637	3.04	0.641	1.86	0.421	0.875	0.4316
	14	8	4.94	0.774	2.66	0.715	1.80	0.518	0.914	0.5547
	28	8	4.98	0.504	2.59	0.559	1.94	0.346	1.055	0.5934
Patients with T2DM										
Placebo	1	3	4.60	0.964	2.83	0.874	1.07	0.115	2.260	1.0817
	14	3	4.37	0.586	2.57	0.404	1.03	0.153	2.527	1.1007
	28	3	5.03	1.266	2.93	0.603	1.13	0.153	3.070	2.4333
Volixibat 10 mg	1	8	4.69	1.037	3.01	0.926	1.25	0.245	1.725	0.5263
	14	8	4.20	1.135	2.46	1.086	1.25	0.214	2.074	0.8015
	28	8	4.68	0.924	2.84	0.890	1.29	0.275	1.933	0.5337

*HDL-C* high-density lipoprotein cholesterol, *LDL-C* low-density lipoprotein cholesterol, *N* number of patients for whom data were available, *SD* standard deviation, *T2DM* type 2 diabetes mellitus

On day 1 (baseline), measurements of faecal bile acids and serum markers were taken before participants received the first dose of study drug; data are from the pharmacodynamic analysis set

#### Glucose metabolism in the T2DM cohort

In patients with T2DM, serum levels of glucose, insulin and C-peptide, and plasma levels of GLP-1, GLP-2 and PYY were assessed pre-dose (day −1) and post-dose (days 14 and 28), in a fasting state (1 h and immediately before an MTT on days −1, 14 and 28) and in response to an MTT (30-min intervals up to 3 h after the MTT on days −1, 14 and 28).

**Fig. 3:**
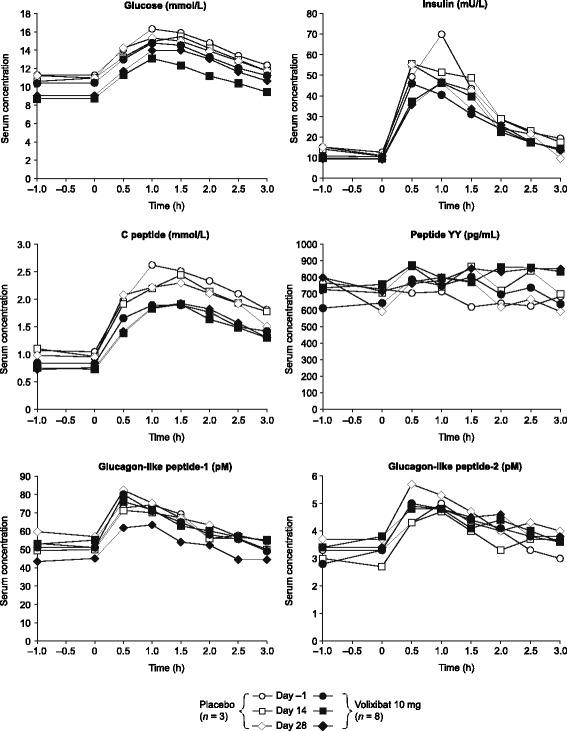
Arithmetic mean concentration–time profiles (pre-meal tolerance test) for glucose metabolism parameters in patients with type 2 diabetes mellitus. Data are from the pharmacodynamic analysis set

In the volixibat group, fasting glucose levels before the MTT (pre-MTT) were lower on days 14 and 28 than those at pre-dose (day −1); pre-MTT glucose levels did not change appreciably between pre-dose and post-dose in the placebo group (Fig. 3). Compared with placebo, least-squares (LS) mean fasting glucose concentrations before the MTT (C_pre_) were nominally significantly lower in the volixibat group on day 14 (−1.8 mmol/L; 90% CI: –2.8, −0.9; *P* = 0.0064) and day 28 (−1.5 mmol/L; 90% CI: –2.5, −0.6; *P* = 0.0163) (Table 5). The glucose response was similar for volixibat and placebo at 0.5–3 h after the MTT (post-MTT), indicating that this was not altered by once-daily volixibat 10 mg administration for 14 or 28 days (Fig. 3). This was confirmed by the E_max_, AUC_(0–3)_ and rAUC_(0–3)_ values for glucose (Additional file 2: Table S1).

**Table 5:** Exploratory comparison of glucose metabolism pharmacodynamic parameters between the volixibat 10 mg and placebo groups in patients with type 2 diabetes mellitus

	Parameter	LS mean value		Difference in LS mean value (volixibat–placebo)	
		Volixibat	Placebo	Mean (90% CI)	*P* value
Glucose					
Day 14	C_pre_ (mmol/L)	8.9	10.7	–1.8 (−2.8, −0.9)	0.0064
	E_max_ (mmol/L)	4.7	4.5	0.2 (−1.8, 2.2)	0.8524
	AUC_(0–3)_ (h.mmol/L)	34.3	39.2	−4.9 (−10.1, 0.3)	0.1173
	rAUC_(0–3)_ (h.mmol/L)	7.6	7.5	0.1 (−4.4, 4.6)	0.9739
Day 28	C_pre_ (mmol/L)	9.2	10.7	−1.5 (−2.5, −0.6)	0.0163
	E_max_ (mmol/L)	5.6	4.4	1.1 (−0.8, 3.1)	0.3147
	AUC_(0–3)_ (h.mmol/L)	37.8	39.6	−1.8 (−7.0, 3.4)	0.5408
	rAUC_(0–3)_ (h.mmol/L)	10.1	7.8	2.3 (−2.2, 6.8)	0.3714
Insulin					
Day 14	C_pre_ (mU/L)	10.0	9.6	0.4 (−3.3, 4.0)	0.8618
	E_max_ (mU/L)	41.3	50.7	−9.4 (−26.9, 8.1)	0.3509
	AUC_(0–3)_ (h.mU/L)	94.2	92.7	1.4 (−24.1, 27.0)	0.9195
	rAUC_(0–3)_ (h.mU/L)	62.9	67.4	−4.5 (−29.8, 20.7)	0.7499
Day 28	C_pre_ (mU/L)	9.6	9.9	−0.3 (−4.0, 3.4)	0.8832
	E_max_ (mU/L)	42.9	39.5	3.5 (−14.0, 20.9)	0.7256
	AUC_(0–3)_ (h.mU/L)	91.8	82.4	9.4 (−16.2, 34.9)	0.5173
	rAUC_(0–3)_ (h.mU/L)	61.8	56.4	5.4 (−19.9, 30.6)	0.7045
C-peptide					
Day 14	C_pre_ (nmol/L)	0.780	0.823	−0.043 (−0.203, 0.117)	0.6313
	E_max_ (nmol/L)	1.407	1.297	0.110 (−0.232, 0.452)	0.5706
	AUC_(0–3)_ (h.nmol/L)	5.002	4.985	0.017 (−0.719, 0.753)	0.9667
	rAUC_(0–3)_ (h.nmol/L)	2.631	2.601	0.030 (−0.826, 0.886)	0.9500
Day 28	C_pre_ (nmol/L)	0.810	0.812	−0.002 (−0.162, 0.158)	0.9797
	E_max_ (nmol/L)	1.407	1.164	0.243 (−0.099, 0.585)	0.2243
	AUC_(0–3)_ (h.nmol/L)	5.181	4.930	0.251 (−0.486, 0.987)	0.5443
	rAUC_(0–3)_ (h.nmol/L)	2.719	2.578	0.141 (−0.715, 0.996)	0.7700
GLP-1					
Day 14	C_pre_ (pM)	55	50	5 (−10, 20)	0.5552
	E_max_ (pM)	23	29	−6 (−16, 4)	0.2731
	AUC_(0–3)_ (h.pM)	191	185	7 (−33, 47)	0.7643
	rAUC_(0–3)_ (h.pM)	26	35	−10 (−35, 16)	0.5128
Day 28	C_pre_ (pM)	45	57	−12 (−27, 3)	0.1894
	E_max_ (pM)	21	27	−6 (−16, 4)	0.2941
	AUC_(0–3)_ (h.pM)	161	200	−39 (−79, 1)	0.1100
	rAUC_(0–3)_ (h.pM)	25	29	−4 (−30, 21)	0.7645
GLP-2					
Day 14	C_pre_ (ngl/mL)	3.80	2.72	1.08 (−0.14, 2.31)	0.1396
	E_max_ (ngl/mL)	1.16	2.15	−0.99 (−1.68, −0.30)	0.0280
	AUC_(0–3)_ (h.ngl/mL)	12.67	12.24	0.43 (−2.06, 2.92)	0.7597
	rAUC_(0–3)_ (h.ngl/mL)	1.46	3.59	−2.14 (−3.89, −0.38)	0.0524
Day 28	C_pre_ (ngl/mL)	3.40	3.84	−0.44 (−1.66, 0.78)	0.5267
	E_max_ (ngl/mL)	1.69	1.84	−0.15 (−0.84, 0.55)	0.7043
	AUC_(0–3)_ (h.ngl/mL)	12.77	14.30	−1.53 (−4.03, 0.96)	0.2886
	rAUC_(0–3)_ (h.ngl/mL)	2.77	2.30	0.47 (−1.28, 2.22)	0.6347
Peptide YY					
Day 14	C_pre_ (pg/mL)	770	668	102 (−173, 377)	0.5141
	E_max_ (pg/mL)	227	270	−43 (−241, 155)	0.6986
	AUC_(0–3)_ (h.pg/mL)	2444	2448	−4 (−563, 555)	0.9897
	rAUC_(0–3)_ (h.pg/mL)	85	578	−493 (−942, −44)	0.0752
Day 28	C_pre_ (pg/mL)	727	555	172 (−103, 447)	0.2808
	E_max_ (pg/mL)	249	353	−105 (−302, 93)	0.3569
	AUC_(0–3)_ (h.pg/mL)	2398	2190	208 (−351, 767)	0.5122
	rAUC_(0–3)_ (h.pg/mL)	167	658	−491 (−941, −42)	0.0760
Glucose–insulin					
Day 14	HOMA2-%B (−)	37.4	26.2	11.2 (0.4, 22.0)	0.0888
	HOMA2-IR (−)	1.5	1.5	0 (−0.6, 0.5)	0.9137
Day 28	HOMA2-%B (−)	35.1	29.6	5.5 (−5.3, 16.3)	0.3724
	HOMA2-IR (−)	1.4	1.5	−0.1 (−0.7, 0.4)	0.6466

*AUC*_*(0–3)*_area under the effect (serum/plasma concentration)–time curve from time 0 to 3 h after the MTT calculated using the linear trapezoidal rule, *CI* confidence interval, *C*_*pre*_ pre-MTT concentration, *E*_max_ maximum observed change from the pre-MTT baseline measurement, *GLP* glucagon-like peptide, *HOMA2-IR* updated homeostasis model assessment of insulin resistance, *HOMA2-%B* homeostasis model assessment of β-cell function, *LS* mean least-squares mean, *MTT* meal tolerance test, *rAUC*_*(0–3)*_ area under the effect (serum/plasma concentration)–time curve from time 0 to 3 h after the MTT calculated using the linear trapezoidal rule with baseline subtracted (rAUC_(0–3)_ = AUC_(0–3)_ – [C_pre_ × 3]) (baseline is the value immediately prior to the standardised breakfast [Ensure Plus®, Abbott Nutrition, Lake Forest, IL, USA])

Comparisons were made between parameters in the volixibat and placebo groups using a mixed model with treatment, study day and treatment-by-study-day interaction as fixed factors, participant within treatment as a random factor, and the day −1 baseline value for each participant as a covariate; data are from the pharmacodynamic analysis set

Fasting, pre-MTT insulin and C-peptide levels were similar in the volixibat and placebo groups before dosing (day −1) and after dosing (days 14 and 28). Post-MTT, there were no consistent or nominally significant differences between the volixibat and placebo groups in LS mean E_max_, AUC_(0–3)_ or rAUC_(0–3)_ for insulin or C-peptide; for C-peptide, there was a trend towards lower values in the volixibat groups compared with the placebo group (Table 5 and Additional file 2: Table S1). There were no obvious changes in fructosamine or HbA_1c_ levels from baseline to day 28.

For both GLP-1 and GLP-2, pre-MTT levels were similar in the volixibat and placebo groups before dosing (day −1) and after dosing (days 14 and 28) (Fig. 3). There were no nominally significant post-MTT differences between the volixibat and placebo groups in LS mean E_max_, AUC_(0–3)_ or rAUC_(0–3)_ for GLP-1 at any time point (Table 5). For GLP-2, E_max_ was nominally significantly lower in the volixibat group than in the placebo group on day 14 (−0.99; 90% CI: –1.68, −0.30; *P* = 0.0280), but not on day 28 (−0.15; 90% CI: –0.84, 0.55; *P* = 0.7043), and there were no differences in AUC_(0–3)_ or rAUC_(0–3)_ on days 14 or 28.

Fasting, pre-MTT PYY levels were not notably different in the volixibat and placebo groups before dosing (day −1) or after dosing (days 14 and 28) (Fig. 3), and there were no nominally significant post-MTT differences in C_pre_, E_max_, AUC_(0–3)_ or rAUC_(0–3)_ at any time point (Table 5 and Additional file 2: Table S1).

In participants receiving volixibat, there was a trend towards an increase in β-cell function (based on values from HOMA2-%B) and a decrease in insulin resistance (based on values from HOMA2-IR) on days 14 and 28 compared with pre-treatment (day −1); in the placebo group, HOMA2-%B and HOMA2-IR values remained relatively constant throughout the study. In the volixibat group, mean (± SD) HOMA2-%B values were higher on day 14 (37.00 ± 7.824) and day 28 (34.68 ± 12.161) than on day −1 (29.18 ± 7.177) (Additional file 3: Table S2); the difference between the volixibat and placebo groups approached nominal significance on day 14 (difference in LS means: 11.2; 90% CI: 0.4, 22.0; *P* = 0.0888) (Table 5). Conversely, mean HOMA2-IR values were lower on day 14 (1.43 ± 0.483) and day 28 (1.35 ± 0.493) than on day −1 (1.64 ± 0.616) in the volixibat group (Additional file 3: Table S2); differences between the volixibat and placebo groups were not nominally significant on days 14 or 28.

## Discussion

In this phase 1 study, inhibition of the ASBT by volixibat at daily doses of 0.5–10.0 mg for 28 days reduced BA reabsorption and substantially increased levels of BA in the colon of HVs and patients with T2DM, as indicated by an increase in faecal BA excretion (a 1.6–3.2-fold increase in HVs and an eightfold increase in patients with T2DM). The elevation in BA excretion was accompanied by increased BA synthesis, as indicated by the rise in serum C4 concentrations in participants receiving volixibat. If these effects are indicative of removal of free cholesterol from the liver through synthesis of new BA from cholesterol, we hypothesise that larger studies of longer duration involving patients with NASH could result in therapeutically beneficial metabolic, anti-inflammatory, anti-steatotic and anti-fibrotic effects.

In a recently published congress abstract of a phase 1 study of volixibat in overweight and obese adults [51], volixibat increased faecal BA and serum C4 levels compared with placebo, indicating that these PD effects are consistent across studies and occur in a patient population that is characteristic of NASH. In this phase 1 study [51], volixibat was administered for 12 days at doses of up to 40 mg daily; doses of at least 20 mg once daily resulted in maximal inhibition of BA reabsorption [51]. In addition, the results of this study demonstrated that the median reduction in total cholesterol level from baseline to the final on-treatment assessment was greater with volixibat than with placebo, and that the median LDL-C level was reduced in all volixibat groups during treatment, compared with a small increase from baseline in the placebo group.

Clinical studies of two other ASBT inhibitors (A4250 and elobixibat; Albireo, Gothenburg, Sweden) have obtained results consistent with those of the current study [38–41, 52]. The authors of a phase 1, placebo-controlled study that included assessment of A4250 treatment for 7 days at doses of 1 mg or 3 mg once daily, or 1.5 mg twice daily, reported that faecal BA excretion was fivefold higher on day 7 in the 3 mg once-daily group than in the placebo group; the magnitude of the treatment effect was less in the 1.5 mg twice-daily group and was similar to that of placebo in the 1 mg once-daily group (no values reported) [52]. The authors also illustrated that plasma C4 levels increased from baseline to day 7 in the A4250 groups (no values reported) [52]. Four placebo-controlled studies of elobixibat in patients with constipation demonstrated consistent increases in plasma C4 levels at once-daily doses of 0.1–20 mg for up to 8 weeks; faecal BA excretion was not assessed in these studies [38–41]. Together with the results of the current study, these findings indicate that ASBT inhibition increases the faecal excretion of BAs and suggest that this may result in upregulation of hepatic BA synthesis, which may help to maintain BA homeostasis in the liver.

Although there was evidence of greater BA synthesis during treatment with volixibat than with placebo in the current study, clinically significant decreases in serum LDL-C levels were not observed. However, in patients with T2DM, those treated with volixibat showed a trend towards increased levels of serum HDL-C, decreased levels of serum triglycerides and reductions in fasting glucose levels, suggesting improvements in both lipid and glucose homeostasis. In the other phase 1 study of volixibat in overweight and obese adults, the median reduction in LDL-C levels over 12 days was 0.6990 mmol/L across the volixibat groups, which included patients treated with fixed doses of up 40 mg daily [51]. Reductions in LDL-C levels were also observed in three [38–40] of the four studies of elobixibat in patients with constipation [41]. Together, these findings are generally consistent with synthesis of new BAs from free cholesterol in the liver to maintain serum BA levels and the hypothesis that increased BA excretion will result in ‘mobilisation’ of harmful lipids from the liver.

In patients with T2DM in the current study, volixibat 10 mg daily did not alter glucose response to the MTT notably, but there was a nominally significant reduction in fasting blood glucose concentration on days 14 and 28. Increases in insulin and C-peptide levels were slightly less with volixibat than with placebo following the MTT. There was also a trend towards improved insulin sensitivity (increasing HOMA2-%B and decreasing HOMA2-IR) in the volixibat group. These PD results are promising, given the short duration of treatment and small sample size – limitations that are inherent to phase 1 studies. To the authors’ knowledge, no other studies of ASBT inhibition in patients with T2DM have been published, although a study of elobixibat in patients with chronic constipation reported that increases in peak GLP-1 levels, but not morning basal or total AUC levels, on day 12 were greater in the active treatment groups (elobixibat 15 mg and 20 mg once daily) than in the placebo group [39]. Previous studies have shown that diabetes, hypertension, dyslipidaemia and obesity are associated with an increased risk of NASH and of advanced fibrosis among patients with NAFLD [53, 54]. Thus, lowering serum and hepatic lipid levels and improving the regulation of metabolic function may be of benefit in this patient population.

Treatment with volixibat at daily doses of 0.5–10 mg for 28 days was generally well tolerated. Overall, the safety and PK profiles of volixibat in this study were consistent with those found in the phase 1 study of volixibat in overweight and obese adults, and are broadly consistent with those reported for other ASBT inhibitors [38–41, 52]. In the present study, there were no deaths, and the one serious TEAE that was reported was considered to be unrelated to the study drug. TEAEs were predominantly gastrointestinal in nature. This was expected given the minimal absorption of volixibat, which results in an increased proportion of BAs reaching the colon. BAs activate intracellular secretory processes in the colon, raising mucosal permeability, increasing mucus secretion and inhibiting chloride hydroxyl exchange [55–57]. In addition, colonic motility is increased as BAs stimulate colonic contractions and reduce colonic transit time [55, 58]. While this mechanism of action resulted in diarrhoea in 36/45 participants receiving volixibat, compared with 4/15 participants in the placebo group, this TEAE was considered to be mild or moderate in severity and generally decreased over time, with none of the events being reported as serious TEAEs. It should also be noted that the composition of serum BA species, including the secondary BAs deoxycholic acid and lithocholic acid, was not altered by volixibat, even though treatment increased total faecal BA excretion by up to eightfold compared with placebo. This finding suggests that treatment does not alter the conditions required for normal biotransformation of primary BAs (predominantly cholic acid and chenodeoxycholic acid) into secondary BAs (deoxycholic acid and lithocholic acid, respectively) by intestinal bacteria.

## Conclusions

In conclusion, daily oral volixibat – a highly potent and selective ASBT inhibitor – was minimally absorbed and generally well tolerated, and increased serum C4 levels and faecal excretion of BAs in HVs and patients with T2DM. In the T2DM cohort, the trends towards increased levels of HDL-C, decreased levels of serum triglycerides and reductions in fasting glucose levels were suggestive of improvements in both lipid and glucose homeostasis. The findings from this study support the initiation of a phase 2 trial (ClinicalTrials.gov Identifier: NCT02787304) to evaluate the efficacy, safety and tolerability of volixibat in adults with NASH.

## Additional files

Additional file 1: Complete inclusion and exclusion criteria. (DOCX 44 kb)

Additional file 2: Table S1. Glucose metabolism parameters in patients with type 2 diabetes mellitus. (DOCX 67 kb)

Additional file 3: Table S2. Glucose–insulin metabolism pharmacodynamic parameters in patients with type 2 diabetes mellitus. (DOCX 43 kb)

## Abbreviations

ASBT: Apical sodium-dependent bile acid transporter
AUC: Area under the plasma concentration–time curve
AUC_(0–3)_: Area under the effect (serum/plasma concentration)–time curve from time 0 to 3 h after the MTT
BA: Bile acids
BMI: Body mass index
C4: 7α-Hydroxy-4-cholesten-3-one
CI: Confidence interval
C_pre_: Pre-meal tolerance test concentration
EBV: Epstein–Barr virus
E_max_: Maximum observed change from the pre-meal tolerance test baseline measurement
FGF: Fibroblast growth factor
FXR: Farnesoid X receptor
GLP: Glucagon-like peptide
HbA_1c_: Glycosylated haemoglobin
HDL-C: High-density lipoprotein cholesterol
HOMA2-%B: Updated homeostasis model assessment of β-cell function
HOMA2-IR: Updated homeostasis model assessment of insulin resistance
HV: Healthy volunteer
LDL-C: Low-density lipoprotein cholesterol
LS: Least-squares
MTT: Meal tolerance test
NAFLD: Non-alcoholic fatty liver disease
NASH: Non-alcoholic steatohepatitis
PD: Pharmacodynamic
PK: Pharmacokinetic
PRA: Pharmaceutical Research Associates
PYY: Peptide YY
SD: Standard deviation
T2DM: Type 2 diabetes mellitus
TEAE: Treatment-emergent adverse event
US: United States
