# Supplementary material for: Safety, tolerability and pharmacodynamics of apical sodium-dependent bile acid transporter inhibition with volixibat in healthy adults and patients with type 2 diabetes mellitus: a randomised placebo-controlled trial
Source: BMC Gastroenterol. 2018 Jan 5;18:3. doi: 10.1186/s12876-017-0736-0 (PMC5756385; doi:10.1186/s12876-017-0736-0)
Supplement: Supplementary file 1 — Complete inclusion and exclusion criteria. (DOCX 44 kb) [file 12876_2017_736_MOESM1_ESM.docx]

## Additional file 1

### Methods Complete inclusion and exclusion criteria

### Inclusion and exclusion criteria

#### Inclusion criteria

Participants were eligible to participate in the study if they met all the following inclusion criteria.

1. Status: healthy volunteers (HVs); patients with type 2 diabetes mellitus (T2DM).

2. Sex: male or female; women could be of childbearing or non-childbearing potential.

3. Age

HVs

Doses 0.5, 1.0, 5.0 mg: 18–55 years, inclusive.

Dose 10 mg: 45–65 years, inclusive.

Patients with T2DM

Dose 10 mg: 18–70 years, inclusive.

4. Body mass index

HVs: 18.0–30.0 kg/m^2^.

Patients with T2DM: 22.0–35.0 kg/m^2^.

5. Women of childbearing potential had to use two of the following acceptable methods of birth control for the times specified below, or had to be sexually inactive (abstinent) from the time of screening until 30 days after the completion of the study or the first menstrual period (whichever was longer).

Intra-uterine device in place for at least 3 months before day 1.

Barrier method (condom or diaphragm) for at least 14 days before screening until study completion.

Stable hormonal contraceptive for at least 3 months before day 1.

Surgical sterilisation (vasectomy) of partner at least 6 months before day 1.

6. Women of non-childbearing potential either were postmenopausal (defined as 1 year of spontaneous amenorrhoea or 6 months of spontaneous amenorrhoea with serum follicle stimulating hormone levels >40 mIU/mL) or had undergone one of the following sterilisation procedures at least 6 months prior to day 1.

Bilateral tubal ligation.

Hysterectomy.

Hysterectomy with unilateral or bilateral oophorectomy.

Bilateral oophorectomy.

7. Men agreed to be sexually abstinent or to use a condom when engaging in sexual activity from admission until completion of the follow-up visit on day 35. Participants were advised to use a condom for 90 days following the last administration of the study drug, and could not donate sperm during this same period of time. In the event that the sexual partner was surgically sterile, use of a condom was not necessary.

8. Regular bowel habits (not more than 3 stools per day or less than one every 2 days).

9. Ability and willingness to abstain from alcohol, methylxanthine-containing beverages or food (coffee, tea, cola, chocolate, ‘power drinks’) and grapefruit (juice) from 48 h before entry into the clinical research centre.

10. Medical history without clinically significant abnormalities.

11. Normal resting supine blood pressures and pulse rate, or showing no clinically relevant deviations as judged by the principal investigator.

12. Computerised (12-lead) electrocardiogram recording without signs of clinically relevant pathology or showing no clinically relevant deviations as judged by the PI.

13. All values for haematology and for clinical chemistry tests of blood and urine within the normal range or showing no clinically relevant deviations as judged by the PI.

14. Agreed to comply with the study protocol and provide written informed consent.

#### Additional inclusion criteria specific to patients with T2DM

15. Diagnosed with T2DM (non-insulin-dependent).

16. Taking a stable dose of one or more oral anti-diabetic medications, such as metformin, sulphonylurea or any other orally administered glucose-lowering medication (except for thiazolidinediones) for at least 3 months prior to screening.

17. Receiving no other long-term medications, including dietary supplements, which significantly alter blood glucose control.

18. Able and willing to wash out all anti-diabetic medication for 14 days prior to dosing.

19. HbA_1c_ (glycosylated haemoglobin) >6.0% and <10% at screening.

20. Fasting blood glucose was within 7.0–12.5 mmol/L, inclusive, at entry into the clinical research centre (day –2).

#### Exclusion criteria

Volunteers were excluded from participation in the study if any of the following exclusion criteria applied.

1. Pregnant women or nursing mothers.

2. For HVs only: a history of any chronic disease.

3. Evidence of uncontrolled, clinically significant cardiovascular, pulmonary, gastrointestinal (including ileal resection, diarrhoea or constipation), renal, hepatic (including cholestasis), pancreatic, haematological, genitourinary, endocrine, infectious, immunological or neurological abnormality in the judgement of the PI.

4. A history of any clinically significant illness within the 3 months prior to screening.

5. A history of cholecystectomy or biliary sphincterotomy.

6. Mental handicap.

7. A history of relevant drug and/or food allergies.

8. A history of substance abuse, drug addiction or alcoholism.

9. Tobacco/nicotine-containing product users for a minimum of 6 months prior to screening.

10. For HVs: use of medication, except for paracetamol (acetaminophen), which was allowed up to 3 days before entry into the clinical research centre. All other medication (including over-the-counter medication, health supplements and herbal remedies such as St John’s Wort extract) had to be stopped at least 14 days before entry to the clinical research centre.

11. Donation or loss of more than 50 mL of blood within 60 days before drug administration. Donation of more than 1.5 L of blood (for men) or more than 1.0 L of blood (for women) in the 10 months preceding the start of the study.

12. Received any investigational medication within 60 days, or within five times the elimination half-life or pharmacodynamic half-life of that drug, whichever was longer, before the first dose of study medication, or was scheduled to receive any investigational medication (other than volixibat) during the course of the study.

13. Positive drug screen (opiates, methadone, cocaine, amphetamines, cannabinoids, barbiturates, benzodiazepines and alcohol).

14. Intake of more than 24 units of alcohol per week (one unit of alcohol equals approximately 250 mL of beer, 100 mL of wine or 35 mL of spirits).

15. Positive screen on hepatitis B surface antigen, anti-hepatitis C virus or anti-human immunodeficiency virus 1/2.

16. Known hypersensitivity to volixibat or other inhibitors of the apical sodium-dependent bile acid transporter.

17. Previously admitted into this study or other studies that administered volixibat.

#### Additional exclusion criteria specific to patients with T2DM

18. The use of insulin and thiazolidinediones for T2DM 3 months before screening was not allowed.

19. Advanced diabetic complications, including neuropathy, nephropathy, retinopathy or other symptoms.
