# Supplementary material for: Safety, tolerability and pharmacodynamics of apical sodium-dependent bile acid transporter inhibition with volixibat in healthy adults and patients with type 2 diabetes mellitus: a randomised placebo-controlled trial
Source: BMC Gastroenterol. 2018 Jan 5;18:3. doi: 10.1186/s12876-017-0736-0 (PMC5756385; doi:10.1186/s12876-017-0736-0)
Supplement: Supplementary file 2 — Glucose metabolism parameters in patients with type 2 diabetes mellitus. (DOCX 67 kb) [file 12876_2017_736_MOESM2_ESM.docx]

**Additional file 2: Table S1** Glucose metabolism parameters in patients with type 2 diabetes mellitus

|  |  |  | Absolute value | | | |  | Absolute change from baseline^a^ | | | |
| --- | --- | --- | --- | --- | --- | --- | --- | --- | --- | --- | --- |
|  | Day | C_pre_ | E_max_ |  | AUC_(0–3)_ | rAUC_(0–3)_ |  | C_pre_ | E_max_ | AUC_(0–3)_ | rAUC_(0–3)_ |
| Glucose |  | (mmol/L) | (mmol/L) |  | (h.mmol/L) | (h.mmol/L) |  | (mmol/L) | (mmol/L) | (h.mmol/L) | (h.mmol/L) |
| Placebo (*n* = 3) | –1 | 11.27 ± 1.365 | 5.07 ± 2.139 |  | 43.17 ± 7.885 | 9.37 ± 3.790 |  |  |  |  |  |
|  |  | (9.8, 12.5) | (3.2, 7.4) |  | (34.7, 50.3) | (5.3, 12.8) |  |  |  |  |  |
|  | 14 | 10.97 ± 0.569 | 4.53 ± 1.350 |  | 41.00 ± 4.850 | 8.10 ± 3.161 |  | –0.30 ± 0.872 | –0.53 ± 0.839 | –2.17 ± 3.523 | –1.27 ± 1.007 |
|  |  | (10.5, 11.6) | (3.2, 5.9) |  | (36.6, 46.2) | (5.1, 11.4) |  | (–0.9, 0.7) | (–1.5, 0.0) | (–4.3, 1.9) | (–2.2, –0.2) |
|  | 28 | 10.97 ± 2.003 | 4.50 ± 1.609 |  | 41.33 ± 7.271 | 8.43 ± 1.537 |  | –0.30 ± 0.656 | –0.57 ± 1.193 | –1.83 ± 1.790 | –0.93 ± 2.887 |
|  |  | (8.9, 12.9) | (3.2, 6.3) |  | (34.4, 48.9) | (7.4, 10.2) |  | (–0.9, 0.4) | (–1.4, 0.8) | (–3.8, –0.3) | (–2.6, 2.4) |
| Volixibat 10 mg | –1 | 10.46 ± 1.676 | 4.81 ± 1.169 |  | 39.24 ± 6.292 | 7.85 ± 2.606 |  |  |  |  |  |
| (*n* = 8) |  | (8.8, 13.6) | (3.6, 6.6) |  | (33.0, 52.4) | (3.9, 11.6) |  |  |  |  |  |
|  | 14 | 8.76 ± 0.784 | 4.66 ± 1.300 |  | 33.66 ± 4.838 | 7.38 ± 3.737 |  | –1.70 ± 1.378 | –0.15 ± 1.581 | –5.58 ± 5.073 | –0.47 ± 3.215 |
|  |  | (8.0, 10.3) | (3.2, 7.0) |  | (25.1, 39.8) | (0.5, 12.8) |  | (–3.3, 0.1) | (–2.0, 2.7) | (–13.5, 2.4) | (–4.0, 5.3) |
|  | 28 | 9.06 ± 1.031 | 5.56 ± 1.819 |  | 37.11 ± 6.141 | 9.93 ± 4.180 |  | –1.40 ± 0.947 | 0.75 ± 2.307 | –2.12 ± 5.151 | 2.08 ± 4.534 |
|  |  | (7.2, 10.4) | (3.9, 9.7) |  | (29.7, 48.7) | (6.8, 19.6) |  | (–3.2, 0.0) | (–0.9, 6.1) | (–10.4, 7.3) | (–2.5, 12.1) |
| Insulin |  | (mU/L) | (mU/L) |  | (h.mU/L) | (h.mU/L) |  | (mU/L) | (mU/L) | (h.mU/L) | (h.mU/L) |
| Placebo (*n* = 3) | –1 | 12.63 ± 4.131 | 61.47 ± 28.980 |  | 114.63 ± 25.408 | 76.73 ± 20.512 |  |  |  |  |  |
|  |  | (10.1, 17.4) | (34.2, 91.9) |  | (85.4, 131.4) | (55.1, 95.9) |  |  |  |  |  |
|  | 14 | 10.77 ± 6.093 | 55.40 ± 24.550 |  | 110.50 ± 41.281 | 78.20 ± 23.059 |  | –1.87 ± 1.963 | –6.07 ± 33.290 | –4.13 ± 31.799 | 1.47 ± 27.062 |
|  |  | (7.1, 17.8) | (34.0, 82.2) |  | (83.3, 158.0) | (62.0, 104.6) |  | (–3.0, 0.4) | (–41.9, 23.9) | (–36.9, 26.6) | (–27.9, 25.4) |
|  | 28 | 11.00 ± 4.838 | 44.13 ± 13.823 |  | 100.17 ± 35.907 | 67.17 ± 21.419 |  | –1.63 ± 0.945 | –17.33 ± 27.823 | –14.47 ± 24.218 | –9.57 ± 23.888 |
|  |  | (7.4, 16.5) | (31.2, 58.7) |  | (72.0, 140.6) | (49.8, 91.1) |  | (–2.7, –0.9) | (–49.4, 0.4) | (–39.2, 9.2) | (–35.3, 11.9) |
| Volixibat 10 mg | –1 | 10.44 ± 3.690 | 45.90 ± 30.352 |  | 85.51 ± 30.057 | 54.20 ± 22.202 |  |  |  |  |  |
| (*n* = 8) |  | (5.2, 16.2) | (11.0, 109.1) |  | (48.1, 135.4) | (22.5, 86.8) |  |  |  |  |  |
|  | 14 | 9.55 ± 3.154 | 39.58 ± 18.244 |  | 87.53 ± 29.804 | 58.88 ± 23.964 |  | –0.89 ± 2.250 | –6.33 ± 17.397 | 2.01 ± 7.512 | 4.68 ± 12.069 |
|  |  | (4.0, 14.9) | (10.7, 73.4) |  | (50.4, 142.2) | (19.9, 97.5) |  | (–5.6, 1.5) | (–35.7, 19.3) | (–9.2, 13.6) | (–5.6, 30.4) |
|  | 28 | 9.13 ± 3.375 | 41.15 ± 16.535 |  | 85.14 ± 24.684 | 57.76 ± 20.988 |  | –1.31 ± 4.024 | –4.75 ± 19.851 | –0.38 ± 19.131 | 3.56 ± 19.888 |
|  |  | (5.3, 15.6) | (16.1, 63.8) |  | (50.0, 118.5) | (29.0, 93.3) |  | (–6.8, 6.2) | (–45.3, 19.3) | (–24.8, 30.9) | (–15.3, 46.5) |
| C-peptide |  | (nmol/L) | (h.nmol/L) |  | (h.nmol/L) | (h.nmol/L) |  | (h.nmol/L) | (h.nmol/L) | (h.nmol/L) | (h.nmol/L) |
| Placebo (*n* = 3) | –1 | 1.046 ± 0.27235 | 1.784 ± 0.50372 |  | 6.487 ± 1.5704 | 3.349 ± 0.76813 |  |  |  |  |  |
|  |  | (0.874, 1.36) | (1.31, 2.31) |  | (5.46, 8.30) | (2.75, 4.22) |  |  |  |  |  |
|  | 14 | 0.9607 ± 0.32020 | 1.553 ± 0.26558 |  | 5.979 ± 1.3745 | 3.097 ± 0.46756 |  | –0.0853 ± 0.05654 | –0.2313 ± 0.34295 | –0.5080 ± 0.37723 | –0.2520 ± 0.53577 |
|  |  | (0.761, 1.33) | (1.34, 1.85) |  | (5.00, 7.55) | (2.63, 3.56) |  | (–0.143, –0.030) | (–0.460, 0.163) | (–0.745, –0.073) | (–0.655, 0.356) |
|  | 28 | 0.9500 ± 0.35531 | 1.420 ± 0.51661 |  | 5.924 ± 1.9874 | 3.074 ± 0.95257 |  | –0.0960 ± 0.08316 | –0.3640 ± 0.04729 | –0.5627 ± 0.42708 | –0.2747 ± 0.20244 |
|  |  | (0.732, 1.36) | (0.962, 1.98) |  | (4.53, 8.20) | (2.26, 4.12) |  | (–0.146, 0.000) | (–0.418, –0.330) | (–0.932, –0.095) | (–0.494, –0.095) |
| Volixibat 10 mg | –1 | 0.8309 ± 0.24610 | 1.263 ± 0.45424 |  | 4.914 ± 1.3818 | 2.421 ± 0.79160 |  |  |  |  |  |
| (*n* = 8) |  | (0.546, 1.31) | (0.618, 2.13) |  | (3.33, 7.76) | (1.19, 3.83) |  |  |  |  |  |
|  | 14 | 0.7281 ± 0.24523 | 1.311 ± 0.43409 |  | 4.630 ± 1.3732 | 2.445 ± 0.84594 |  | –0.1028 ± 0.07533 | 0.0478 ± 0.25556 | –0.2841 ± 0.29431 | 0.0241 ± 0.47211 |
|  |  | (0.407, 1.26) | (0.532, 1.86) |  | (3.07, 7.16) | (0.944, 3.38) |  | (–0.242, –0.004) | (–0.270, 0.412) | (–0.600, 0.300) | (–0.450, 1.03) |
|  | 28 | 0.7585 ± 0.18995 | 1.312 ± 0.33894 |  | 4.809 ± 1.0517 | 2.533 ± 0.84269 |  | –0.0724 ± 0.16994 | 0.0486 ± 0.32381 | –0.1051 ± 0.79159 | 0.1120 ± 0.82683 |
|  |  | (0.487, 1.12) | (0.759, 1.75) |  | (3.56, 6.52) | (1.16, 4.09) |  | (–0.332, 0.122) | (–0.380, 0.562) | (–1.24, 0.877) | (–0.665, 1.87) |
| GLP-1 |  | (pM) | (pM) |  | (h.pM) | (h.pM) |  | (pM) | (pM) | (h.pM) | (h.pM) |
| Placebo (*n* = 3) | –1 | 51.0 ± 32.74 | 27.7 ± 16.92 |  | 191.3 ± 120.74 | 38.3 ± 23.69 |  |  |  |  |  |
|  |  | (15, 79) | (9, 42) |  | (56, 288) | (11, 53) |  |  |  |  |  |
|  | 14 | 50.0 ± 30.32 | 28.7 ± 5.13 |  | 185.7 ± 95.01 | 35.7 ± 4.16 |  | –1.0 ± 9.54 | 1.0 ± 13.00 | –5.7 ± 34.59 | –2.7 ± 19.73 |
|  |  | (15, 68) | (23, 33) |  | (76, 243) | (31, 39) |  | (–11, 8) | (–12, 14) | (–45, 20) | (–16, 20) |
|  | 28 | 57.0 ± 37.40 | 26.0 ± 12.12 |  | 200.7 ± 114.27 | 29.7 ± 18.15 |  | 6.0 ± 14.80 | –1.7 ± 4.93 | 9.3 ± 21.73 | –8.7 ± 28.59 |
|  |  | (14, 82) | (13, 37) |  | (69, 274) | (13, 49) |  | (–4, 23) | (–5, 4) | (–14, 29) | (–40, 16) |
| Volixibat 10 mg | –1 | 51.0 ± 16.56 | 33.4 ± 15.41 |  | 189.9 ± 58.69 | 36.9 ± 17.02 |  |  |  |  |  |
| (*n* = 8) |  | (26, 78) | (13, 58) |  | (94, 288) | (7, 54) |  |  |  |  |  |
|  | 14 | 55.1 ± 26.78 | 23.1 ± 5.89 |  | 191.1 ± 66.37 | 25.8 ± 20.78 |  | 4.1 ± 11.86 | –10.3 ± 17.91 | 1.3 ± 25.32 | –11.1 ± 26.11 |
|  |  | (26, 103) | (15, 33) |  | (117, 298) | (–11, 55) |  | (–11, 25) | (–43, 8) | (–40, 30) | (–65, 20) |
|  | 28 | 45.1 ± 21.19 | 20.8 ± 9.13 |  | 160.5 ± 63.99 | 25.0 ± 23.53 |  | –5.9 ± 12.26 | –12.6 ± 15.36 | –29.4 ± 38.59 | –11.9 ± 23.81 |
|  |  | (25, 88) | (1, 28) |  | (91, 292) | (–12, 44) |  | (–26, 10) | (–32, 6) | (–85, 18) | (–61, 15) |
| GLP-2 |  | (ng/mL) | (ng/mL) |  | (h.ng/mL) | (h.ng/mL) |  | (ng/mL) | (ng/mL) | (h.ng/mL) | (h.ng/mL) |
| Placebo (*n* = 3) | –1 | 3.263 ± 1.0456 | 1.797 ± 0.7057 |  | 12.107 ± 3.6781 | 2.317 ± 0.5443 |  |  |  |  |  |
|  |  | (2.06, 3.95) | (0.99, 2.30) |  | (7.88, 14.58) | (1.70, 2.73) |  |  |  |  |  |
|  | 14 | 2.720 ± 1.3301 | 2.117 ± 0.2003 |  | 11.673 ± 4.3779 | 3.513 ± 0.8801 |  | –0.543 ± 0.6035 | 0.320 ± 0.5533 | –0.433 ± 1.2186 | 1.197 ± 0.6726 |
|  |  | (1.40, 4.06) | (1.91, 2.31) |  | (6.92, 15.54) | (2.72, 4.46) |  | (–1.08, 0.11) | (–0.17, 0.92) | (–1.30, 0.96) | (0.63, 1.94) |
|  | 28 | 3.840 ± 1.7579 | 1.807 ± 0.5150 |  | 13.737 ± 4.9841 | 2.217 ± 0.3150 |  | 0.577 ± 0.7966 | 0.010 ± 0.2951 | 1.630 ± 1.7459 | –0.103 ± 0.8558 |
|  |  | (1.93, 5.39) | (1.29, 2.32) |  | (8.37, 18.22) | (2.02, 2.58) |  | (–0.13, 1.44) | (–0.29, 0.30) | (0.49, 3.64) | (–0.68, 0.88) |
| Volixibat 10 mg | –1 | 3.260 ± 1.4795 | 2.130 ± 0.9211 |  | 12.775 ± 4.1037 | 2.995 ± 1.3106 |  |  |  |  |  |
| (*n* = 8) |  | (1.86, 6.60) | (0.79, 3.16) |  | (8.59, 21.88) | (1.03, 4.69) |  |  |  |  |  |
|  | 14 | 3.799 ± 2.1976 | 1.176 ± 0.5833 |  | 12.883 ± 5.5553 | 1.486 ± 1.5929 |  | 0.539 ± 1.1789 | –0.954 ± 0.9996 | 0.110 ± 2.3334 | –1.508 ± 1.8981 |
|  |  | (1.49, 8.64) | (0.51, 2.16) |  | (7.44, 25.11) | (–0.81, 3.69) |  | (–0.49, 2.65) | (–2.12, 0.91) | (–2.83, 3.41) | (–4.55, 1.96) |
|  | 28 | 3.396 ± 1.6805 | 1.708 ± 0.5902 |  | 12.984 ± 4.7285 | 2.795 ± 1.3538 |  | 0.136 ± 0.8488 | –0.423 ± 1.0308 | 0.211 ± 1.9422 | –0.198 ± 1.7712 |
|  |  | (2.01, 7.06) | (0.95, 2.82) |  | (7.76, 23.00) | (1.43, 5.55) |  | (–1.39, 1.53) | (–2.16, 0.90) | (–3.86, 1.77) | (–2.82, 2.61) |
| Peptide YY |  | (pg/mL) | (pg/mL) |  | (h.pg/mL) | (h.pg/mL) |  | (pg/mL) | (pg/mL) | (h.pg/mL) | (h.pg/mL) |
| Placebo (*n* = 3) | –1 | 727.3 ± 337.49 | 102.7 ± 38.28 |  | 2002.0 ± 939.64 | –180.0 ± 80.88 |  |  |  |  |  |
|  |  | (492, 1114) | (60, 134) |  | (1316, 3073) | (–269, –111) |  |  |  |  |  |
|  | 14 | 704.7 ± 236.32 | 194.0 ± 47.62 |  | 2367.3 ± 557.26 | 253.3 ± 233.83 |  | –22.7 ± 111.61 | 91.3 ± 46.36 | 365.3 ± 484.92 | 433.3 ± 154.02 |
|  |  | (506, 966) | (158, 248) |  | (1794, 2907) | (9, 475) |  | (–148, 66) | (42, 134) | (–166, 784) | (278, 586) |
|  | 28 | 592.0 ± 200.03 | 277.3 ± 165.96 |  | 2109.7 ± 259.31 | 333.7 ± 504.20 |  | –135.3 ± 510.00 | 174.7 ± 131.79 | 107.7 ± 946.45 | 513.7 ± 583.57 |
|  |  | (394, 794) | (128, 456) |  | (1858, 2376) | (–6, 913) |  | (–720, 218) | (68, 322) | (–978, 759) | (105, 1182) |
| Volixibat 10 mg | –1 | 643.3 ± 190.08 | 227.8 ± 152.77 |  | 2204.0 ± 450.58 | 274.1 ± 361.82 |  |  |  |  |  |
| (*n* = 8) |  | (390, 896) | (80, 564) |  | (1287, 2687) | (–149, 1051) |  |  |  |  |  |
|  | 14 | 755.8 ± 228.23 | 255.5 ± 191.60 |  | 2474.3 ± 410.59 | 207.0 ± 431.56 |  | 112.5 ± 117.55 | 27.8 ± 157.92 | 270.3 ± 216.65 | –67.1 ± 308.06 |
|  |  | (434, 1196) | (44, 614) |  | (1871, 3042) | (–546, 914) |  | (–38, 300) | (–184, 222) | (–86, 584) | (–545, 452) |
|  | 28 | 713.3 ± 293.59 | 277.3 ± 198.25 |  | 2428.6 ± 684.21 | 288.9 ± 488.36 |  | 70.0 ± 213.57 | 49.5 ± 148.50 | 224.6 ± 533.72 | 14.8 ± 239.48 |
|  |  | (432, 1288) | (58, 620) |  | (1317, 3305) | (–559, 1091) |  | (–236, 434) | (–84, 378) | (–793, 891) | (–410, 424) |

^a^Baseline is the day –1 value

*AUC_(0–3)_* area under the effect (serum/plasma concentration)–time curve from time 0 to 3 h after the MTT, *C_pre_* pre-MTT concentration, *E_max_* maximum observed change from the pre-MTT baseline measurement, *GLP* glucagon-like peptide, *MTT* meal tolerance test, *rAUC_(0–3)_* area under the effect (serum/plasma concentration)–time curve from time 0 to 3 h after the MTT calculated using the linear trapezoidal rule with baseline subtracted (rAUC_(0–3)_ = AUC_(0–3)_ – [C_pre_ × 3]) (baseline is the value immediately prior to the standardised breakfast [Ensure Plus^®^, Abbott Nutrition, Lake Forest, IL, USA])

Values are mean ± standard deviation (minimum, maximum); data are from the pharmacodynamic analysis set
