# Supplementary material for: Safety, tolerability and pharmacodynamics of apical sodium-dependent bile acid transporter inhibition with volixibat in healthy adults and patients with type 2 diabetes mellitus: a randomised placebo-controlled trial
Source: BMC Gastroenterol. 2018 Jan 5;18:3. doi: 10.1186/s12876-017-0736-0 (PMC5756385; doi:10.1186/s12876-017-0736-0)
Supplement: Supplementary file 3 — Glucose–insulin metabolism pharmacodynamic parameters in patients with type 2 diabetes mellitus. (DOCX 43 kb) [file 12876_2017_736_MOESM3_ESM.docx]

**Additional file 3: Table S2** Glucose–insulin metabolism pharmacodynamic parameters in patients with type 2 diabetes mellitus

|  |  | Absolute value | |  | Absolute change from baseline | |
| --- | --- | --- | --- | --- | --- | --- |
| Glucose–insulin | Day | HOMA2-%B | HOMA2-IR |  | HOMA2-%B | HOMA2-IR |
| Placebo (*n* = 3) | –1 | 30.73 ± 14.674 | 1.97 ± 0.551 |  |  |  |
|  |  | (20.9, 47.6) | (1.6, 2.6) |  |  |  |
|  | 14 | 27.27 ± 13.745 | 1.67 ± 0.896 |  | –3.47 ± 1.002 | –0.30 ± 0.346 |
|  |  | (18.4, 43.1) | (1.1, 2.7) |  | (–4.5, –2.5) | (–0.5, 0.1) |
|  | 28 | 30.63 ± 20.436 | 1.70 ± 0.624 |  | –0.10 ± 5.813 | –0.27 ± 0.115 |
|  |  | (17.8, 54.2) | (1.2, 2.4) |  | (–3.8, 6.6) | (–0.4, –0.2) |
| Volixibat 10 mg (*n* = 8) | –1 | 29.18 ± 7.177 | 1.64 ± 0.616 |  |  |  |
|  |  | (18.3, 40.2) | (0.8, 2.7) |  |  |  |
|  | 14 | 37.00 ± 7.824 | 1.43 ± 0.483 |  | 7.83 ± 9.498 | –0.21 ± 0.327 |
|  |  | (22.8, 47.3) | (0.6, 2.3) |  | (–6.2, 24.4) | (–0.8, 0.1) |
|  | 28 | 34.68 ± 12.161 | 1.35 ± 0.493 |  | 5.50 ± 9.033 | –0.29 ± 0.651 |
|  |  | (23.7, 53.2) | (0.8, 2.3) |  | (–4.3, 20.2) | (–1.3, 0.9) |

*HOMA2-IR* updated homeostasis model assessment of insulin resistance, *HOMA2-%B* updated homeostasis model assessment of β-cell function

Values are mean ± standard deviation (minimum, maximum) and are calculated using fasting glucose and insulin concentrations as determined by the HOMA2 calculator of the University of Oxford (Version 2.2.2) for the pre-meal-tolerance-test sample; data are from the pharmacodynamic analysis set
